# Tamoxifen-Induced Epigenetic Silencing of Oestrogen-Regulated Genes in Anti-Hormone Resistant Breast Cancer

**DOI:** 10.1371/journal.pone.0040466

**Published:** 2012-07-10

**Authors:** Andrew Stone, Fatima Valdés-Mora, Julia M. W. Gee, Lynne Farrow, Richard A. McClelland, Heidi Fiegl, Carol Dutkowski, Rachael A. McCloy, Robert L. Sutherland, Elizabeth A. Musgrove, Robert I. Nicholson

**Affiliations:** 1 Welsh School of Pharmacy, Redwood Building, Cardiff University, Cardiff, Wales, United Kingdom; 2 The Kinghorn Cancer Centre and Cancer Research Program, Garvan Institute of Medical Research, Sydney, New South Wales, Australia; 3 Department of Epigenetics, Garvan Institute of Medical Research, Sydney, New South Wales, Australia; 4 Department of Gynaecology and Obstetrics, Innsbruck Medical University, Innsbruck, Austria; 5 St Vincent’s Clinical School, Faculty of Medicine, University of New South Wales, St Vincent’s Hospital, Sydney, New South Wales, Australia; The University of Arizona, United States of America

## Abstract

In the present study, we have taken the novel approach of using an *in vitro* model representative of tamoxifen-withdrawal subsequent to clinical relapse to achieve a greater understanding of the mechanisms that serve to maintain the resistant-cell phenotype, independent of any agonistic impact of tamoxifen, to identify potential novel therapeutic approaches for this disease state. Following tamoxifen withdrawal, tamoxifen-resistant MCF-7 cells conserved both drug resistance and an increased basal rate of proliferation in an oestrogen deprived environment, despite reduced epidermal growth-factor receptor expression and reduced sensitivity to gefitinib challenge. Although tamoxifen-withdrawn cells retained ER expression, a sub-set of ER-responsive genes, including pS2 and progesterone receptor (PgR), were down-regulated by promoter DNA methylation, as confirmed by clonal bisulphite sequencing experiments. Following promoter demethylation with 5-Azacytidine (5-Aza), the co-addition of oestradiol (E2) restored gene expression in these cells. In addition, 5-Aza/E2 co-treatment induced a significant anti-proliferative effect in the tamoxifen-withdrawn cells, in-contrast to either agent used alone. Microarray analysis was undertaken to identify genes specifically up regulated by this co-treatment. Several anti-proliferative gene candidates were identified and their promoters were confirmed as more heavily methylated in the tamoxifen resistant vs sensitive cells. One such gene candidate, growth differentiation factor 15 (GDF15), was carried forward for functional analysis. The addition of 5-Aza/E2 was sufficient to de-methylate and activate GDF15 expression in the tamoxifen resistant cell-lines, whilst in parallel, treatment with recombinant GDF15 protein decreased cell survival. These data provide evidence to support a novel concept that long-term tamoxifen exposure induces epigenetic silencing of a cohort of oestrogen-responsive genes whose function is associated with negative proliferation control. Furthermore, reactivation of such genes using epigenetic drugs could provide a potential therapeutic avenue for the management of tamoxifen-resistant breast cancer.

## Introduction

Despite the obvious benefit tamoxifen has provided for millions of oestrogen receptor alpha (ER) positive breast cancer patients worldwide, almost all patients with metastatic disease and as many as 40% of patients receiving adjuvant tamoxifen will acquire resistance to the drug’s inhibitory effect on breast cancer cell proliferation [1], [2]. Originally it was thought that the acquisition of resistance was caused by a loss or mutation of the ER, as is often the case in patients with intrinsic anti-hormone resistance [2], [3]. However, it has since been shown that breast cancer cells that have lost anti-oestrogen sensitivity often retain an ER positive phenotype with normal ER functionality [4], [5]. In tamoxifen resistant breast cancer, ER interacts with deregulated growth-factor pathways, facilitating resistant cell proliferation [2], [6]. This cross-talk can be targeted with other hormonal therapies such as the pure anti-oestrogen fulvestrant [7], which depletes ER. However, it too is subject to the subsequent development of resistance mechanisms [8], whilst growth-factor pathway blockade (e.g. gefitinib) has also proven relatively disappointing in the clinic to date [9], suggesting further key mechanisms underlie anti-hormone resistant growth.

Recent literature suggests that although the interruption of ER-signalling by long-term tamoxifen exposure does not appear to deplete ER-expression, it can induce epigenetic modifications to ER-regulated gene promoters, leading to sustained alterations in phenotype [10]. Tamoxifen-bound ER is recruited to oestrogen response elements within a target gene promoter, much the same as oestrogen-bound ER. The inhibitory effect on ER-regulated gene transcription caused by tamoxifen is the result of conformational changes within the ligand binding domain of the ER that provide docking sites for co-repressors of transcriptional activity, including NCoR/SMRT [11], REA [12], RTA [13], SAFB1 [14] and SMAD4 [15]. As part of their inhibitory function, these co-repressors recruit histone de-acetylases (HDACs) to the receptor complex which serve to modify the chromatin environment surrounding the promoter to which the complex is bound. For example, during tamoxifen-induced suppression of the classically regulated oestrogen responsive gene, pS2, the time course of recruitment of the HDAC complexes precisely coincides with that of the deacetylation of histone H3 and H4 tails at the target promoter, providing crucial support for the hypothesis that tamoxifen functions as an antagonist in breast cells by inducing epigenetic modification [16]. Significantly, gene promoters suppressed in such a manner are vulnerable to more permanent epigenetic changes, such as promoter methylation [17]. DNA-methyl transferase DNMT-1 and DNMT3a/DNMT3b bind HDAC2 and HDAC1, respectively, to achieve effective gene silencing [18], [19]. A study by Fan et al (2006) showed that 75% of genes that were up-regulated in oestradiol-treated parental MCF-7 breast cancer cells (≥2 fold increase in expression), were no longer inducible in a tamoxifen-resistant sub-line, highlighting the potential scale of this phenomenon [20].

Although several studies have shown that long-term tamoxifen treatment can induce distinct global gene expression and promoter DNA methylation profiles in breast cancer cells [10], [20], it is unclear how this event might contribute to anti-hormone resistant cell proliferation. Interestingly, long-term tamoxifen treatment of MCF-7 cells decreased levels of several pro-apoptotic genes and impaired the subsequent apoptotic response to etoposide treatment [21]. Another study conducted by Wu et al (2007) used SAGE analysis to show that the expression of the tumour suppressor gene retinoblastoma binding protein 8 (CtIP) was decreased in models of acquired tamoxifen resistance, where its knockdown in MCF-7 cells promoted tamoxifen resistance, and induction restored response [22]. Promoter methylation of the tumour suppressor genes identified in these studies was not examined; however, such data suggest that long-term tamoxifen treatment may induce genotypic changes that contribute to anti-hormone resistant cell proliferation.

The research question addressed in the present study, therefore, was whether ER-regulated genes associated with an anti-proliferative function were silenced by promoter methylation in tamoxifen-resistant breast cancer cells as a consequence of prolonged tamoxifen treatment; and whether re-activation of such genes could induce an anti-proliferative response and thus provide a potential therapeutic avenue for the management of tamoxifen-resistant disease.

## Materials and Methods

### Cell Culture

ER-positive, MCF-7 breast cancer cells, given to our laboratory by AstraZeneca (Cheshire, UK), were maintained in RPMI-1640 based medium containing 5% (v/v) FCS, antibiotics (streptomycin (10 µg/ml), penicillin (10 IU/ml), fungizone (2.5 µg/ml) (RPMI+5%). All tissue culture media and constituents were purchased from Invitrogen and plastic-ware was obtained from Nunc. Tamoxifen-resistant MCF-7 (TAM-R) cells were generated by the long-term culture of MCF-7 cells in phenol-red-free RPMI medium containing 5% charcoal stripped FCS and 4-OH-tamoxifen (1×10^−7^ M) (TAM) (Sigma-Aldrich) as previously described [23]. In the present study, the TAM-R cells were maintained in TAM-free medium for up to 6 months to produce tamoxifen-withdrawn TAM-R cell sub-lines (1, 3 and 6 month withdrawal). Throughout this study TAM-Wd refers to TAM-R cells withdrawn from tamoxifen for 6 months unless otherwise stated. The authenticity of all cell-lines was verified by STR profiling (CellBank Australia, Westmead, NSW, Australia).

### Proliferation and Dose Response Analysis

Cells were seeded into 24-well plates at a density of 1×10^4^ cells/well. After 24 hrs, the treatments were added. Dose response experiments using 4-OH tamoxifen (1×10^−10^ M to 1×10^−6^ M), gefitinib (AstraZeneca) (1×10^−9^ M to 1×10^−6^ M) and oestradiol (Sigma-Aldrich) (E2; 1×10^−12^ M to 1×10^−7^ M) in the presence of 5-azacytidine (Sigma-Aldrich) (1×10^−6^ M) and/or 4-OH tamoxifen (1×10^−7^ M) were terminated after a further 6-days of culture. Dose response experiments using recombinant GDF-15 protein (R&D Systems) (1, 3, 10 ng/ml) were terminated after 48 hrs. Proliferation assays using fulvestrant (AstraZeneca) (1×10^−7^ M), gefitinib (1×10^−6^ M) or 5-Azacytidine (1×10^−6^ M) in combination with E2 (1×10^−9^ M) and/or 4-OH tamoxifen (1×10^−7^ M) were monitored for up to 14 days. Cell medium was typically changed every 3 days and all cell counts were determined by coulter counter analysis. Data shown represent the findings of 3 independent experiments.

### RT-qPCR

The present study used the Applied Biosystems High Capacity R.T Kit and the DyNAmo™ SYBR Green qPCR Kit (Finnzymes). RT was carried out on RNA harvested from MCF-7, TAM-R and TAM-Wd cells 72 hrs post-seeding on to 10 cm dishes (±E2−1×10^−9^ M) and cells treated with E2 (1×10^−9^ M), 5-Azacytidine (1×10^−6^ M), 5-Aza + E2 and 5-Aza + E2+4-OH tamoxifen (1×10^−7^ M) for 48-hrs post-treatment. The expression of the following genes was analysed; ER (5′-ggagacatgagagctgccaac-3′ and 3′-ccagcagcagcatgtcgaagatc-5′), EGFR (5′-caacatctccgaaagcca-3′ and 3′-cggaactttgggcgactat-5′), pS2 (5′-catggagaacaaggtgatctg-3′ and 3′-cagaagcgtgtctgaggtgtc-5′), PgR (5′- ccatgtggcagatcccacaggagtt -3′ and 3′- tggaaattcaacactcagtgcccgg -5′), GDF15 (5′-tcccgggaccctcagagt-3′ and 3′-caggtcctcgtagcgtttcc-5′) and β-actin (5′- ggagcaatgatcttgatctt -3′ and 3′- ccttcctgggcatggagtcct -5′).

**Figure 1:**
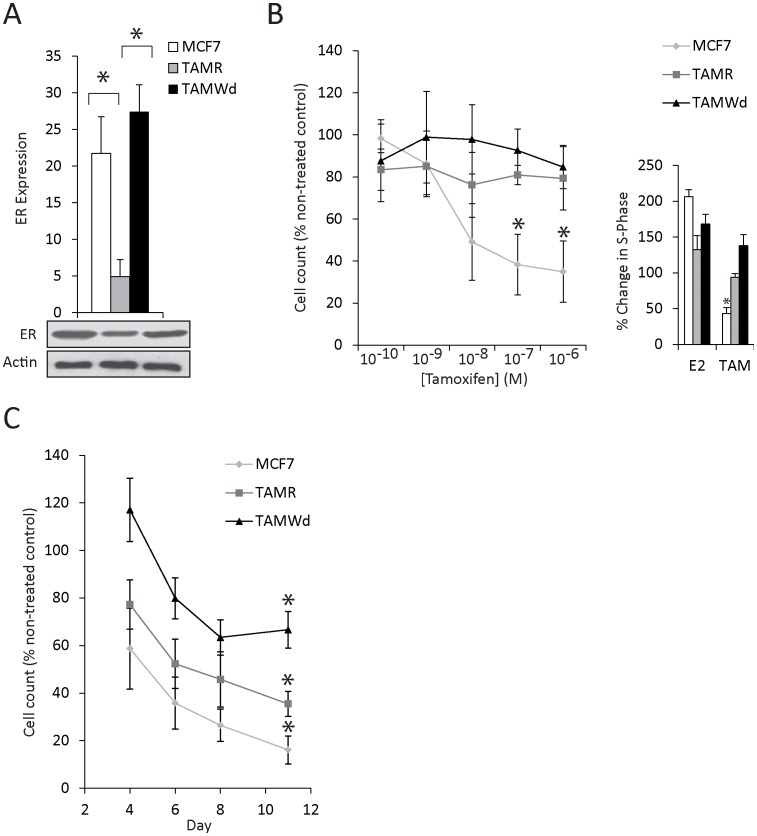
Tamoxifen resistant cells retain ER expression and TAM-resistance following withdrawal from the drug. A. RT-qPCR evaluation of the basal total ER mRNA expression in MCF-7, TAM-R in the presence of TAM) and TAM-Wd cells. Data are shown as arbitrary units after normalisation to actin (n = 3) (*p<0.001). Lysates were western blotted for total ER protein and β-actin (loading control). Data are representative of 3 independent experiments. B. MCF-7, TAM-R and TAM-Wd cell concentration response to TAM (1×10^−10 ^M to 1×10^−6^ M). Cell number was assessed after 7 days. The data shown represent the percentage cell number relative to non-treated control cells (*p<0.001). The bar graph depicts flow cytometry data was used to determine the percentage of cells in S-phase following TAM treatment (1×10^−7^ M). A significant reduction in S-phase was observed only in the MCF-7 cells (*p<0.001). C. Cell proliferation inhibition in response to fulvestrant (1×10^−7^ M). Cell counts were taken on days 4, 6, 8 and 11. The data shown represent the cell number relative to time-matched non-treated control cells. Cell counts for all cell-lines treated with fulvestrant were significantly reduced compared to vehicle treated controls by day 11 of culture (*p<0.001).

### Immunocytochemistry

Cells were seeded onto 22-mm^2^, 3-amino-propyltriethoxysilane coated glass coverslips in 35 mm culture dishes at a density of 1×10^5^ cells/ml and incubated for 24 hrs before treatment (±5-Aza (1×10^−6^ M) ±E2 (1×10^−9^ M) for 48 hrs). Coverslips were fixed and primary antibody was applied (pS2 and PgR - NovaCastra). Staining was visualised with DAKO EnVision™ + system-HRP-labelled polymer and DAKO Liquid DAB+ substrate. Coverslips were counter-stained with methyl-green and visualised at 10× magnification for evaluation of pS2/PgR staining and photographed.

### Western Blotting

Briefly, cell lysate samples (40 µg) were denatured, subjected to electrophoresis separation on SDS polyacrylamide gel and trans-blotted onto a 0.2 µM nitro-cellulose membrane. Blots were blocked then incubated with primary antibody (ER- 1D5 DAKO/EGFR- sc-03-G Upstate Biotechnology/GDF15- Cell Signalling #8479). Following incubation with the appropriate secondary antibody, detection was performed by applying a thin film of enhanced chemiluminescence reagent (Supersignal™). Blots shown reflect the observations from three independent experiments and were standardised for equivalence of loading using β-actin detection.

### Microarray Analysis

Total RNA was extracted from TAM-Wd cells ±5-Aza (1×10^−6^ M) ±E2 (1×10^−9^ M) ±4-OH tamoxifen (1×10^−7^ M) for 48 hrs. Extraction was performed using Tri-reagent (Sigma-Aldrich) and subsequently DNase treated before quantifying by spectrophotometry. Three experimental replicates were performed for each treatment. 5 µg of RNA per cell-subline/treatment was sent for each of the triplicates to Central Biotechnology Services, Cardiff University, who performed RNA integrity analysis, reverse-transcription, labelling and hybridisation to HG-U133A.2 Affymetrix® gene chips. Following scanning of each chip and capture of the expression data with MAS 5.0 software, the data were subsequently uploaded and analysed using Genesifter™ software (www.geospiza.com) after median normalisation and log-transformation. Statistical interrogation of the expression profiles was then performed within Genesifter™ to select all those most highly expressed in 5-Aza/E2 treated cells vs non-treated, E2 or 5-Aza alone and 5-Aza/E2+4-OH tamoxifen treated cells.

### MeDIP-coupled PCR

MeDIP was carried out on DNA extracted from MCF-7, TAM-R and TAM-Wd cells using the MethylMiner™ Methylated DNA Enrichment Kit (Invitrogen) according to the manufacturer’s instructions. QPCR was performed to quantify the abundance of the following genes; PEG3 (5′-gattggcacgtcacagggct-3′ and 3′-gcctcccaaacctctcctcc-5′), GAPDH (5′-tcgacagtcagccgcatct-3′ and 3′-ctagcctcccgggtttctct-5′), RASAL1 (5′-gcccttctgcctggaaagtt-3′ and 3′-ccaccacgcgaacattcag-5′), DUSP7 (5′-ctttctcggcacgattcga-3′ and 3′-ccatcaacaggaaaaaaaaggaa-5′), ATP2B4 (5′-aggctcagagtgcagctattcc-3′ and 3′- ccaacatcgacccctaatcaga-5′) and GDF15 (5′-tcccgggaccctcagagt-3′ and 3′-caggtcctcgtagcgtttcc-5′). Gene expression is presented relative to the hemi-methylated, imprinted gene, PEG3.

**Figure 2:**
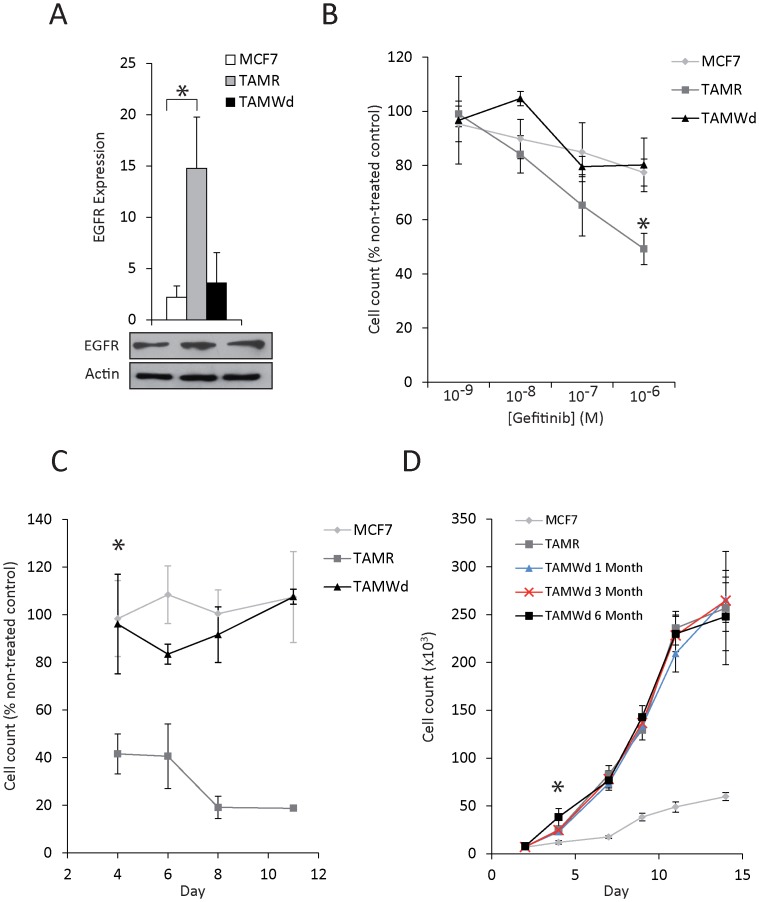
Tamoxifen withdrawal from resistant cells reduces EGFR expression and gefitinib sensitivity, but not the rate of proliferation. A. RT-qPCR evaluation of the basal total EGFR mRNA expression in MCF-7, TAM-R and TAM-Wd cells. Data have been normalised to actin (n = 3) (*p = 0.012). The lower panel shows a western blot of total EGFR protein and β-actin (loading control) (representative of 3 independent experiments). B. MCF-7, TAM-R and TAM-Wd cell concentration response to gefitinib (1×10^−9^ M to 1×10^−6^ M). Cell number was assessed after 7 days. The data shown represent the cell number relative to non-treated control cells. Cell counts for gefitinib treated (1×10^−6^ M) TAM-R cells are significantly lower than vehicle treated controls (*p<0.001). C. Inhibition of cell proliferation in response to gefitinib (1×10^−6^ M). Cell counts were taken on days 4, 6, 8 and 11. TAM-R cells were significantly inhibited by gefitinib challenge from day 4 of culture (*p<0.001). The data represent the cell number relative to time-matched non-treated control cells D. Anchorage-dependent proliferation assay of non-treated MCF-7, TAM-R, and TAM-Wd cells (1, 3 and 6 months withdrawn). Data shown represent actual cell number/well recorded over 3 independent experiments. Counts recorded for all resistant cell-lines were greater than MCF-7 cell counts from day 4 of culture (*p<0.001).

### Clonal Bisulphite Sequencing

Clonal bisulphite sequencing was used to analyse the methylation status of the promoter regions of three different genes; pS2, PgR and GDF15. The bisulphite reaction was carried out on up to 1.5 µg of extracted genomic DNA for 12 hr at 55°C, under conditions previously described in [24]–[26]. After bisulphite conversion, the DNA was precipitated and resuspended in water to a final concentration of 40 ng/µl. Bisulphite converted DNA was then analysed by bisulphite PCR analysis. Triplicate PCR amplifications were performed using the following bisulphite conversion primers: pS2 FW: 5′-gttggtatgaatagttaaaagtattattttgagatttt-3′; pS2 RV: 5′- aaaaaaacaataaccaccataaaaaacaaaata-3′; PgR FW: 5′-tttttygttttgtataggatgtattttag-3′; PgR RV:5′- aaaatcrccctaataaaacaaaa-3′; GDF15 FW:5′- tggtttttagatgtttttggtgttgtt-3′; GDF15 RV:5′- aatttcccaaatactatacacattcaaaaaaa-3′. PCR conditions were optimised as previously described [25], [26]. The triplicates of the PCR products of each condition were pooled to ensure representative clonal analysis and then cloned and sequenced. After cloning and sequencing the methylation state of the individual clones was analysed using BiQ Analyzer software tabulated in a bisulphite map, to visualize the heterogeneity of methylation.

### Flow Cytometry

Cell cycle analysis was achieved by flow cytometric analysis of propidium iodide-stained, ethanol fixed cells. Apoptotic cell populations were determined by the detection of M30 positivity (FITC-conjugated M30 CytoDEATH monoclonal antibody, Alexis Biochemicals). All samples were run on a BD FACSCanto II (BD Biosciences).

### Statistics

The statistical significance of results obtained when comparing between cell-lines or when comparing treated cells versus control counterparts was derived using an independent, two-tailed Student’s t-test. Where multiple data points were present, data were analysed using ANOVA with post-hoc tests (p<0.05 was deemed statistically significant).

**Figure 3:**
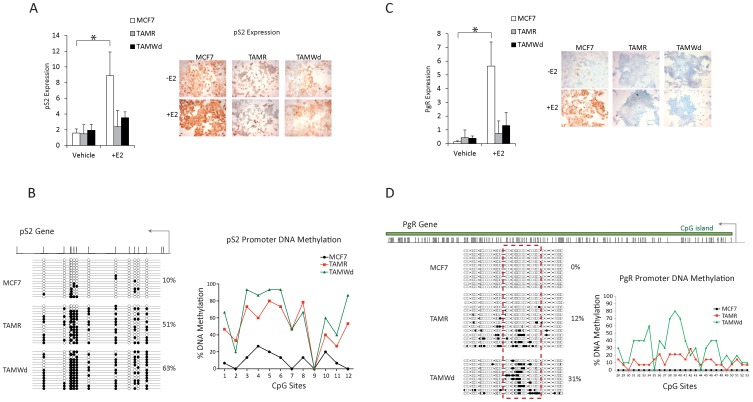
pS2 and PgR are silenced by DNA-methylation in tamoxifen-resistant cells. RT-qPCR evaluation of pS2 (A) and PgR (C) mRNA expression in MCF-7, TAM-R and TAM-Wd cells ± E2 (1×10^−9^ M) for 48 hrs. Data was normalised to actin (n = 3). Gene expression is significantly induced following E2 challenge in the MCF-7 cells (*p<0.001). The right panel of the figure shows parallel ICC for pS2 (A) and PgR (B) protein (10× magnification) (representative of 3 independent experiments). Clonal bisulphite sequencing analysis was performed across pS2 (B) and PgR (D) gene promoter regions in MCF7, TAM-R and TAM-Wd. PS2 (B) and PgR (D) promoter region maps are shown on the left panel. The PCR amplicons (441 bp for pS2 and 566 bp for PgR) interrogated a total number of 12 CpG sites pS2 gene (B) and 53 CpG sites for PgR (D) located downstream the TSS. Bisulphite maps determined by direct sequencing of individual clones show the density of methylated CpG site (black circle) and unmethylated CpG site (white circle) at individual CpG sites. The line plots on the right panel show the percentage of methylation of each CpG site interrogated in each cell line for pS2 (B) and PgR (D) genes. The lines on the gene promoter maps represent CpG sites and the arrow the Transcriptional Start Site (TSS). The dashed red box denotes the genomic region with the major changes in DNA methylation that was further analyzed in the line plot.

## Results

In order to provide a cellular model of tamoxifen-resistance that was representative of tamoxifen-withdrawal subsequent to clinical relapse, TAM-R cells [23] which are routinely maintained in 4-OH tamoxifen (TAM) (1×10^−7^ M), were cultured in its absence for 6 months. The rationale was that the permanent heritable changes acquired following long-term TAM exposure would be maintained following this period, and any agonistic contribution to cell proliferation would be depleted.

**Figure 4:**
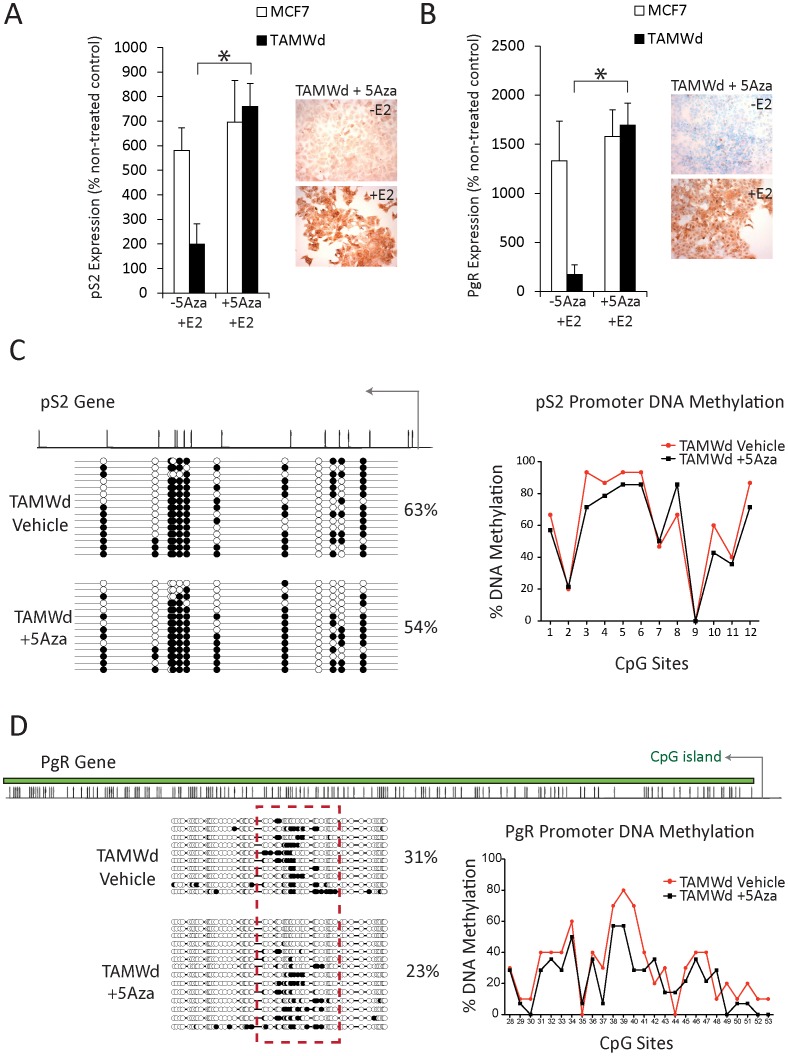
5-Aza/E2 Co-treatment restored pS2 and PgR sensitivity to E2 challenge in the TAM-Wd cells. A. RT-qPCR evaluation of pS2 expression in MCF-7 and TAM-Wd cells ±5-Aza (1×10^−6^ M) ±E2 (1×10^−9^ M) for 48 hrs. Data shown represent percentage increase in pS2 detected in cells ±5-Aza following E2 challenge. Expression of pS2 is significantly increased in TAM-Wd cells treated with 5-Aza/E2 compared to 5-Aza treated cells (*p<0.001). Figure also shows ICC parallel analysis of pS2 protein expression in TAM-Wd cells (10× magnification) (representative of 3 independent experiments). B. RT-qPCR evaluation of PgR expression in MCF-7 and TAM-Wd cells ±5-Aza (1×10^−6^ M) ±E2 (1×10^−9^ M) for 48 hrs. Data shown represent percentage increase in PgR detected in cells ±5-Aza following E2 challenge. Expression of PgR is significantly increased in TAM-Wd cells treated with 5-Aza/E2 compared to 5-Aza treated cells (*p<0.001). Figure also shows ICC parallel analysis of PgR protein expression in TAM-Wd cells (10× magnification) (representative of 3 independent experiments). C. Clonal bisulphite sequencing analysis for the pS2 promoter region in TAM-Wd cells ±5-Aza (1×10^−6^ M) for 48 hrs. Colours and symbols are the same as in Figure 3. D. Clonal bisulphite sequencing analysis for the PgR promoter region in TAM-Wd cells ±5-Aza (1×10^−6^ M) for 48 hrs. Colours and symbols are the same as in Figure 3.

First we determined whether TAM-induced suppression of ER expression was a permanent feature of the TAM-R phenotype. In TAM-R cells in the presence of TAM, ER mRNA and protein expression were significantly reduced compared to the parental MCF-7 cells, although cells remained ER-positive (Fig. 1a) (*p<0.001). Tamoxifen withdrawal restored ER expression to the level detected in the MCF-7 cells (Fig. 1a). Despite the restoration of ER expression in the tamoxifen-withdrawn (TAM-Wd) cells, resistance to TAM was conserved following the 6-month withdrawal period (Fig. 1b). Whilst 6-day treatment with TAM at a concentration of 1×10^−7^ M (or greater) significantly reduced MCF-7 cell counts by 60% (compared to non-treated controls) (*p<0.001), TAM-R and TAM-Wd cell counts were not significantly affected. This is concordant with a significant reduction of MCF-7 cells in the S-phase of the cell cycle following TAM treatment (*p<0.001); an affect that was not observed in TAM-treated TAM-R and TAM-Wd sub-lines (Fig. 1b). The pure ER antagonist fulvestrant (1×10^−7^ M), however, was able to significantly inhibit TAM-Wd cell proliferation, as well as the MCF-7 and TAM-R cell proliferation, consistent with previous observations [27] (Fig. 1c) (*p<0.001). Importantly, the extent to which fulvestrant inhibited proliferation varied between cell lines: TAM-Wd cells were less sensitive than TAM-R which were in turn less sensitive than parental cells. This implies that the significance of ER-signalling as a contributor to cell proliferation decreased following the acquisition of tamoxifen resistance and further decreased following the withdrawal of the drug, although as in the clinical setting, some responsiveness to alternative anti-hormonal challenge clearly remains [7]. In the TAM-R cells, and in the clinical setting, this phenomenon has been closely associated with the emergence of alternative proliferative pathways, including EGFR signalling [23], [28]. We therefore next determined whether the TAM-Wd cells demonstrated continued reliance on EGFR signalling.

**Figure 5:**
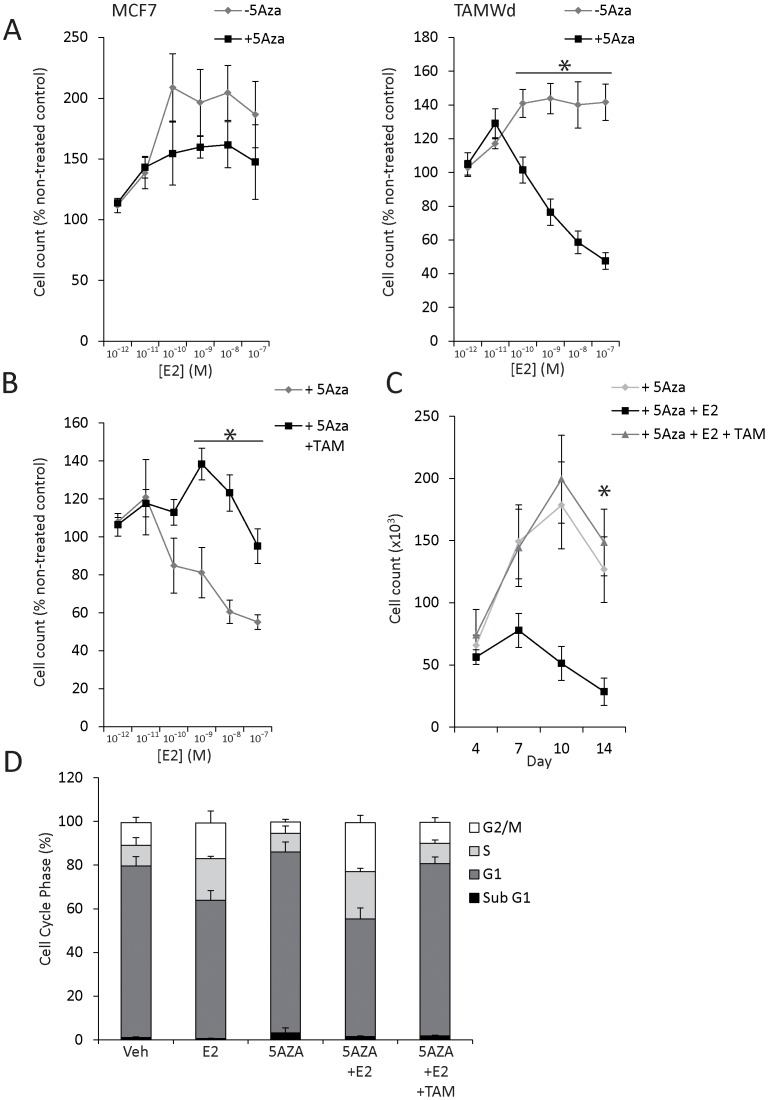
5-Aza/E2 inhibits TAM-Wd cell proliferation. A. TAM-Wd and MCF-7 cell concentration response to E2 challenge (1×10^−12^ M to 1×10^−7^ M) ±5-Aza (1×10^−6^ M). Cell counts were taken on day 7 of culture. Data shown represent E2-treated cell counts as a percentage of non-E2 treated control cells. Cell counts are significantly lower in 5-Aza treated TAM-Wd cells, co-treated with E2 at a concentration of 1×10^−10^ M and greater (*p<0.017). B. TAM-Wd cell concentration response to E2 challenge (1×10^−12^ M to 1×10^−7^ M) +5-Aza (1×10^−6^ M) ±TAM (1×10^−7^ M). Cell counts were taken on day 7. Data shown represent E2-treated cell counts as a percentage of non-E2 treated control cells. The co-addition of TAM to 5-Aza treated TAM-Wd cells significantly changes the effect of E2 from a concentration of 1×10^−9^ M and greater (*p<0.026). C. Anchorage-dependent proliferation assay of TAM-Wd cells treated with E2 (1×10^−9^ M) ± TAM (1×10^−7^ M) in the presence of 5-Aza (1×10^−6^ M) for 14 days. The data shown represent actual cell number/well recorded over 3 independent experiments. By day 14, there are significantly more cells in the 5-Aza and 5-Aza/E2/TAM treated cells compared to TAM-Wd +5-Aza/E2 treated cells (*p<0.001). D. Cell cycle analysis using flow cytometric analysis of propidium iodide-stained TAM-Wd cells. Data represent percentage of cell in each phase relative to the total population.

As expected, EGFR expression in the TAM-R cells (in the presence of TAM) was significantly increased from that expressed in the parental MCF-7 cells (Fig. 2a) (p = 0.012). In contrast, in the TAM-Wd cells, EGFR expression was comparable to the parental cell line (Fig. 2a). When challenged with increasing concentrations of EGFR inhibitor, gefitinib, the highly EGFR-positive TAM-R cells showed a significantly greater (albeit incomplete) growth inhibitory response (∼50%), compared to the MCF-7 and TAM-Wd cells, which were largely insensitive (Fig. 2b). When challenged with gefitinib at a concentration of 1×10^−6^ M, the TAM-Rs demonstrate significantly greater sensitivity (compared to both MCF-7 and TAM-Wd cells) from day 4 of culture (Fig. 2c) (*p<0.001). Therefore, tamoxifen withdrawal caused a reduction in EGFR expression which directly correlated with a loss of sensitivity to gefitinib. However, despite reduced expression of this major driver of resistant cell proliferation, TAM-Wd cells continued to proliferate at a much greater rate than the MCF-7 cells, in a similar fashion to the TAM-R cell-line (Fig. 2d). This led us to investigate whether TAM exposure had a more permanent effect on the genes it suppresses, rather than activates, and whether this could be associated with the permanently enhanced rate of proliferation.

To explore whether ER gene targets could be permanently suppressed following long-term TAM exposure, the expression of the classically regulated genes, pS2 and progesterone receptor (PgR), was assessed by RT-qPCR and ICC in all cell-lines in the presence and absence of E2 (Fig. 3a and 3c). Both pS2 and PgR gene expression were significantly up-regulated in the MCF-7 cells following E2 challenge (*p<0.001), in contrast to the TAM-R and TAM-Wd cells, where no significant increase was detected. ICC showed a concurrent trend at the protein level (Fig. 3a and 3c).

**Figure 6:**
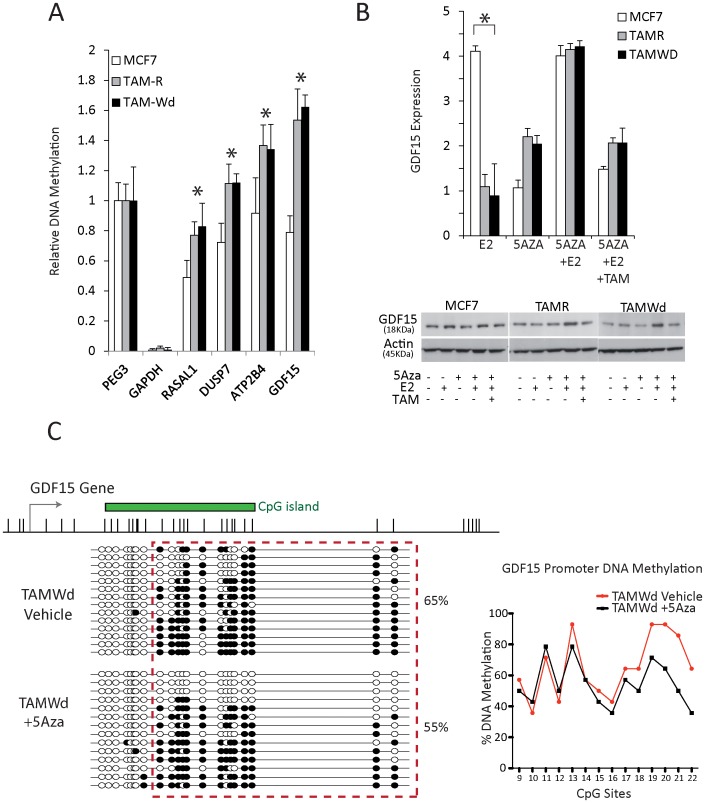
Genes associated with an anti-proliferative function are silenced by DNA methylation in tamoxifen-resistant cells. A. MeDIP coupled PCR to determine the methylation status of RASAL1, DUSP7, ATP2B4 and GDF15 in MCF-7, TAM-R and TAM-Wd cells, relative the expression of PEG3 (an imprinted gene). Data is expressed as 2^∧^−ΔΔct (n = 3). GAPDH is included as a negative control to demonstrate the successful enrichment of methylated material. The data demonstrate a greater enrichment of methylated DNA in resistant vs tamoxifen-sensitive MCF-7 cells for all four genes (*p<0.05). B. RT-qPCR evaluation of GDF15 expression in MCF-7, TAM-R and TAM-Wd cells +E2 (1×10^−9^ M), +5-Aza (1×10^−6^ M), 5-Aza + E2 or 5-Aza + E2+ TAM (1×10^−7^ M) for 48 hrs. Data shown are normalised to GAPDH and presented relative to the expression calculated for vehicle-treated cells. In the absence of 5-Aza, GDF15 expression is only significantly increased by E2 challenge in MCF-7 cells (*p<0.001). The lower panel shows a western blot of mature, processed GDF15 protein (18 KDa) and β-actin (loading control) (representative of 3 independent experiments). C. Clonal bisulphite sequencing analysis for the GDF15 promoter region in TAM-Wd cells ±5-Aza (1×10^−6^ M) for 48 hrs. Colours and symbols are the same as in Figure 3.

**Figure 7:**
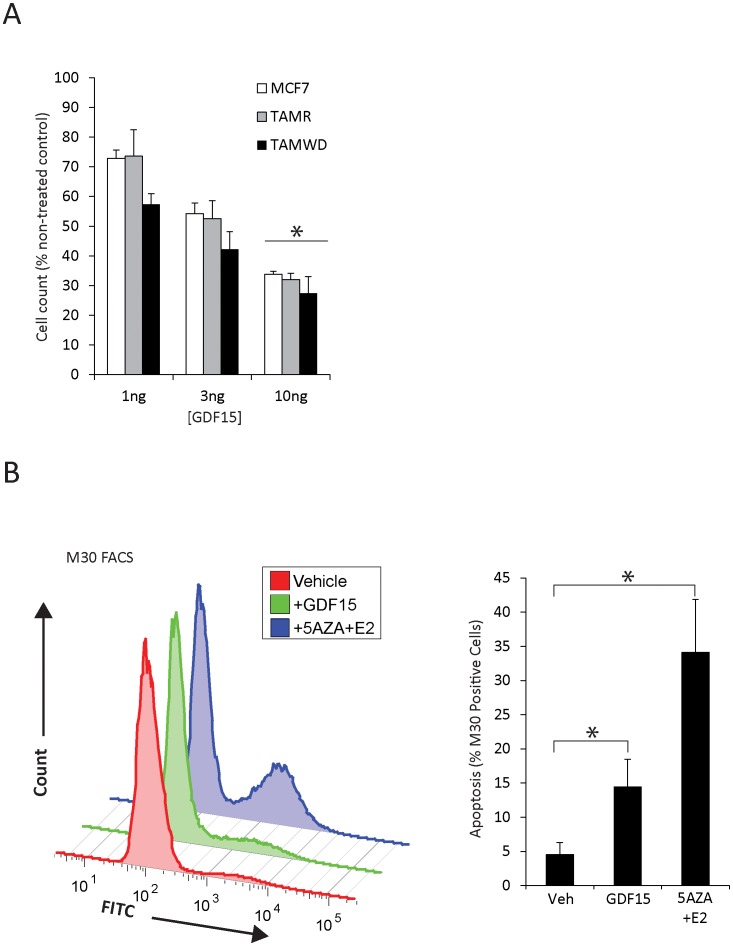
Recombinant GDF15 is anti-proliferative and induces apoptosis in the TAM-Wd cells. A. Dose response to recombinant GDF-15 protein (1, 3, 10 ng/ml) in MCF-7, TAM-R and TAM-Wd cells. Cell number was assessed after 48 hrs post-treatment. The data shown represent the cell number relative to non-treated control cells. The dose-dependent anti-proliferative effect was statistically significant from 1 ng/ml across all cell-lines (*p<0.001). B. Both the addition of recombinant GDF15 and 5-Aza/E2 caused an increase in apoptotic cells as determined by flow cytometric analysis of M30 bound, TAM-Wd cells. Increases in apoptosis were significant for both treatments (*p<0.001), although 5-Aza/E2 induced a greater response.

To determine whether promoter DNA-methylation was the mechanism by which pS2 and PgR were inactivated in the resistant cells, clonal bisulphite sequencing experiments were carried out in non-E2 stimulated MCF-7, TAM-R and TAM-Wd cells (Fig. 3b and 3d). Interestingly, very low levels of pS2 promoter methylation were observed in the MCF-7 cells (10%), in contrast to the more heavily methylated promoter regions of the TAM-R (51% methylated) and TAM-Wd (63% methylated) cell-lines (Fig. 3b). Similarly, none of the CpG residues in the PgR promoter were methylated in the MCF-7 cells, in contrast to the more heavily methylated PgR promoters in the TAM-R (10%) and TAM-Wd (31%) cell-lines (Fig. 3d). Together these data provide proof of principle that decreased E2-sensitivity at a ER-regulated promoter region is associated with increased DNA-methylation in the resistant cell-lines.

MCF-7 and TAM-Wd cells were exposed to the de-methylation agent, 5-Azacytidine (5-Aza) in combination with E2 to investigate whether demethylation could restore pS2 and PgR activation in the resistant cells (Fig. 4a and 4b). Used at a concentration (1 µM) and length of time (48 hrs) previously shown to provide effective DNA de-methylation with minimal cell-cytotoxicity [29], [30], 5-Aza had no effect on the induction of pS2 or PgR in MCF-7 cells. However, in the TAM-Wd cells, 5-Aza restored pS2 and PgR activation in response to E2 as assessed by RT-qPCR and ICC (Fig. 4a and 4b) (*p<0.001).

Clonal bisulphite sequencing was used to analyse the methylation status of the pS2 and PgR promoters in 5-Aza treated TAM-Wd cells (Fig. 4c and 4d). The percentage of methylated CpG residues in the pS2 and PgR gene promoters was reduced from 63% to 54% and from 31% to 23% respectively. Although complete promoter demethylation was not observed in all clones, these findings are consistent with previous reports that suggest gene expression can be restored following partial demethylation of the promoter region [31]–[33]. In this instance, the restoration of gene expression may have been facilitated by other epigenetic modifiers (e.g. histone acetylases) recruited to the site of transcription by the active ER/E2 complex [16], [34]. Interestingly, it is apparent that some clones are more sensitive to 5-Aza induced demethylation. For example, the first 3 clones are completely de-methylated across all CpGs in the PgR promoter following 5-Aza treatment (Fig. 4d), and the first 2 clones are reduced to one single methylated CpG in the pS2 promoter (Fig. 4c).

Surprisingly, 5-Aza treatment also enabled E2, a modest mitogen in the absence of this agent, to become a significant inhibitor of TAM-Wd cell proliferation (Fig. 5a, 5b and 5c). This was apparent with concentrations of E2 from 1×10^−10^ M to 1×10^−7^ M (*p≤0.017) (Fig. 5a). This event was specific to the TAM-Wd cells as parallel experiments in the MCF-7 cells showed that 5-Aza had no effect on E2 response (Fig. 5a). Further analysis showed that the co-addition of TAM (1×10^−7^ M) to TAM-Wd cells cultured with 5-Aza and E2 reversed the inhibition of proliferation (Fig. 5b and 5c), thus supporting the hypothesis that the genes reactivated by 5-Aza/E2 co-treatment in the TAM-Wd cells were ER-dependent. Over a 14 day period, it was apparent that the 5-Aza/E2 treated cells were undergoing cell death from day 7 of culture, whilst the 5-Aza or 5-Aza/E2/TAM treated cells continued to proliferate (Fig. 5c). Fig. 5d shows that the addition of 5-Aza to vehicle or E2 treated cells did not affect TAM-Wd cell cycle phase distribution. Instead, 5-Aza/E2 inhibition of TAM-Wd cells was associated with apoptosis, as suggested by the growth curve (Fig. 5c) and confirmed by M30 flow cytometry (see Fig. 7b).

Microarray analysis of TAM-Wd cells was conducted to identify genes that were up-regulated by E2 challenge only in the presence of the demethylation agent i.e. were most highly expressed in 5-Aza/E2 treated cells, vs non-treated, E2 or 5-Aza-alone treated and 5-Aza/E2+ TAM treated cells (n = 159 probes). Using a second microarray database [35] we also identified genes that were E2 activated in the MCF-7 cells and suppressed in the TAM-R cells, and were thus potentially silenced by promoter methylation following long-term tamoxifen exposure (202 probes p<0.001) *(the gene selection process is summarised in Figure S1).* The 43 probes (34 genes, Table S1) common to both gene sets were high-confidence potential contributors to the altered control of proliferation in the TAM-Wd cells. Using the UCSC Genome browser (http://genome.ucsc.edu/), we determined that of these 34 genes, 23 had bona-fide CpG islands in their promoter regions [36], 9 of which had ER binding sites [37] (Fig. S1 and Table S1).

Many of the genes identified through these analyses were involved in the regulation of several distinct processes that affect cell proliferation and/or apoptosis, including regulators of signal-transduction (EPS15R, RASAL-1 and DUSP-7), calcium transportation (ATP2B4), p53 activation (GDF15 and UNC5B) and hormone metabolism (CYP1B1), all of which have CpG islands in their promoter regions. Using MeDIP-coupled qPCR, we determined 4 of the 7 candidates (RASAL1, DUSP7, ATP2B4 and GDF15) were more heavily methylated in the resistant cell lines vs the parental MCF-7 cells (Fig. 6a) (*p<0.05), with GDF15 showing the greatest difference. Further experiments therefore focussed on GDF15.

RT-qPCR was used to demonstrate that GDF15 expression was regulated by E2 in the MCF-7 cells (*p<0.001), but not the TAM-R and TAM-Wd cell-lines (Fig. 6b). However, with 5-Aza/E2 co-treatment, GDF15 expression increased 4-fold in the resistant cell-lines, similar to the degree of induction in the parental MCF-7s (Fig. 6b). The induction of GDF15 protein in 5-Aza/E2 treated resistant cells was confirmed by western blotting for the processed, mature form of GDF15 in cell lysate (Fig. 6b).

Clonal bisulphite sequencing analysis confirmed that 5-Aza treatment led to demethylation of the GDF15 promoter, with an overall reduction in methylation from 65% to 55% (Fig. 6c). Interestingly, 3 out of the 14 clones showed complete demethylation of the interrogated CpG sites upon 5-Aza treatment (Fig. 6c).

Having shown that active GDF15 was induced following 5-Aza/E2 treatment, dose-response experiments with recombinant GDF15 protein were performed to determine the effect on cell proliferation/apoptosis (Fig. 7a and 7b). Cell counts for GDF15 (10 ng/ml) treated MCF-7, TAM-R and TAM-Wd cells were all significantly lower than vehicle treated control cells (Fig. 7a) (*p<0.001).

Using the M30 CytoDEATH antibody (that detects caspase-cleaved cytokeratin 18 [38]), flow cytometric analysis showed that both GDF15 (10 ng/ml) and 5-Aza/E2 treatment (48 hrs) induced a significant apoptotic response in the TAM-Wd cells (*p<0.001) (Fig. 7b). The percentage of M30-positive cells was increased by 10% following GDF15 exposure, and 30% with 5-Aza/E2 compared to vehicle treated controls (Fig. 7b). Although the magnitude of response was different, the data indicate that both treatments induce the same inhibitory response in the TAM-Wd cells.

## Discussion

Cumulatively, the data from this study provide evidence to support the novel concept that long-term tamoxifen exposure induces epigenetic silencing of oestrogen-responsive genes potentially associated with negative cellular control, which serves to promote resistant cell proliferation. As such, when co-treated with the demethylation agent 5-Azacytidine, we observed that resistant cell growth was markedly inhibited by oestrogen challenge in parallel with the re-expression of such gene cohorts.

The TAM-R cells used in the present study retain ER-expression and function (as indicated by partial responsiveness to further anti-hormone challenge with fulvestrant), and yet in contrast to the parental cells, classically regulated oestrogen-responsive genes, such as pS2 and PgR, are insensitive to oestrogen activation. This was observed in parallel with an increase in pS2/PgR promoter DNA-methylation, demonstrating that ER-signalling disruption caused by long-term tamoxifen exposure can result in a permanent epigenetic modification to the promoters of ER-responsive genes. Critically, pS2 and PgR suppression was maintained following tamoxifen withdrawal from the TAM-R cells, whilst promoter methylation increased, probably due to the extended period of culture in an oestrogen free-environment and thus supressed ER-activity. Interestingly, disease recurrence following adjuvant tamoxifen treatment has been closely associated with the development of an ER positive/PgR negative phenotype in some patients [5] . Our studies raise the possibility that the loss of PgR in these patients could have been due to DNA-promoter methylation and that PgR methylation could be potential bio-marker of tamoxifen insensitivity.

The discrete demethylation at gene promoters induced by 5-Aza in TAMR-Wd cells was sufficient to re-express pS2, PgR and GDF15 expression upon E2 treatment. Other researchers have also demonstrated dramatic increases in the expression of hyper-methylated genes in 5-Aza treated cells, with only partial demethylation of the CpG-rich promoter regions [31]–[33]. It is noteworthy that the clonal bisulphite sequencing data shows that the effect of 5-Aza treatment was heterogeneous, suggesting that demethylation may not occur evenly in cell culture, with some cells being more vulnerable than others. Although our results indicate that the level of methylation across these gene promoters is crucial to re-activating silenced genes, we cannot rule out dependence or crosstalk with other epigenetic factors to induce gene expression. Previous reports demonstrate that active ER recruits co-activators of transcription, often with intrinsic histone-acetylase (HAT) activity, which serve to further adapt the chromatin landscape to facilitate gene transcription [16], [34]. As epigenetic mechanisms often work in synergy to elicit the ultimate response, it is possible that the combination of 5-Aza and HAT activity permitted the observed dramatic increases in pS2, PgR and GDF15 gene expression.

In addition to 5-Aza treatment restoring ER-regulated gene promoter sensitivity to E2, it switched oestradiol from being a modest mitogen to a significant growth inhibitory agent. This previously unidentified phenomenon appeared to be ER related, since it was reversible by tamoxifen co-addition and was not evident in the parental MCF-7 cells, hence confirming it was a consequence of long-term tamoxifen treatment. We hypothesised that in the TAM-Wd cells, demethylation enhances the oestrogen responsiveness of genes that can individually or collectively inhibit proliferation and/or induce apoptosis. Although this may initially appear incongruent, since oestrogens are mitogenic to many breast cancer cell-lines, inhibitory effects of oestrogen are not unprecedented. Oestrogen induces apoptosis in oestrogen-deprived [39], [40] and ER-negative breast cancer cells stably transfected with ER [41], [42]. Furthermore, high doses of synthetic oestrogens, such as diethylstilbestrol (DES), have been used effectively to treat postmenopausal women with ER-positive breast cancer, and cause tumour regression [43]; indicating that under certain circumstances, oestrogen is able to promote anti-proliferative or pro-apoptotic gene networks. Since the TAM-Wd cells remain tamoxifen resistant, it is apparent that they harbour some alterations in oestrogen signalling, and this could make the cells less able to overcome the effects of oestrogen-responsive anti-proliferative genes than the parental MCF-7 cells.

Microarray analysis was used to identify genes that may potentially contribute to the inhibitive effect 5-Aza/E2 treatment had on TAM-Wd cell proliferation, i.e. genes that were E2-responsive prior to long-term tamoxifen treatment, depleted following the acquisition of resistance and readily expressed following 5-Aza/E2 challenge. Some of these candidate genes have ER-binding sites but it is probable that many lie downstream of directly ER-regulated gene, since oestrogenic control of breast cancer cell proliferation and survival is achieved through the regulation of multiple gene networks and signalling pathways, many of which are not directly activated by ER [44]. Although not all candidates identified possess known CpG islands within their promoter region (as is the case for pS2) they may still be subject to epigenetic silencing (caused by DNA methylation) as it has recently become clear that CpG methylation can occur outside of gene promoters and in the absence of CpG islands [45].

GDF-15 is a p53 target gene that inhibits tumour cell growth via the TGFβ signalling pathway [46], [47] and is subject to promoter hypermethylation in renal cancer cells [48]. It inhibits the proliferation of prostate and breast cancer cells, induces colon and mammary epithelial cancer cell apoptosis *in-vitro* and inhibits colon and glioblastoma tumour growth in vivo [46], [49]–[53]. The GDF-15 promoter was more heavily methylated in the tamoxifen-resistant cell-lines compared to the parental MCF-7 cell-line, and 5-Aza/E2 caused promoter demethylation and increased GDF15 expression. Furthermore, recombinant GDF15 protein inhibited cell proliferation in a dose-dependent manner, demonstrating that genes reactivated by 5-Aza/E2 could cause the associated anti-proliferative/apoptotic effect. Many of the other candidate genes identified by our analysis also have functions associated with the control of processes known to contribute to cancer cell proliferation and survival, including regulators of various signalling pathways including EGFR (EPS15R [54]), Ras (RASAL-1 [55]) and ERK (DUSP7 [56]), calcium transportation (ATP2B4 [57], [58]), p53 activation (UNC5B [59]) and hormone metabolism (CYP1B1 [60]).

The potential for anti-hormone-induced epigenetic modification of ER-regulated genes to affect cell proliferation has been clearly demonstrated in this study. It remains unclear as to whether gene promoters become methylated as a direct consequence of sustained tamoxifen binding or whether as a consequence of long-term ER-signalling deprivation (as demonstrated by ER siRNA studies [61]). We have provided evidence that components of multiple regulatory mechanisms, including inhibitors of growth-factor signalling and mediators of p53 function are silenced following long-term tamoxifen treatment in a cellular model. However, more detailed analysis will be required to fully understand the contribution of epigenetic silencing of these genes to tamoxifen resistance. Importantly, this process is reversible with 5-Aza/E2 co-treatment in parallel with a growth inhibitory effect, highlighting a previously unidentified therapeutic opportunity in tamoxifen-resistant breast cancer. Further exploration of this phenomenon in other models of anti-hormone resistance and in clinical disease is warranted.

## Supporting Information

Figure S1 **The microarray gene selection process and Venn diagram depicting ER-binding site/CpG island characteristics of the final gene candidates.** The figure depicts the microarray interrogation strategy used to identify genes that could be associated with the inhibitory effect of 5-Aza/E2 co-treatment in the TAM-Wd cells (shown in Fig. 5a, 5b and 5c). Firstly, genes were selected that were most highly up-regulated in TAM-Wd cells +5-Aza/E2 vs non-treated TAM-Wd cells (n = 744 p<0.05). This gene set was further analysed to identify those genes more highly expressed in 5-Aza/E2 vs 5-Aza treated cells, to ensure the selected genes were oestrogen responsive (n = 240 p<0.05). Since we showed that TAM co-addition could block the inhibitory effect of 5-Aza/E2 treatment (Fig. 5b and 5c), we then selected genes whose expression were reduced in 5-Aza/E2+TAM treated cells vs 5-Aza/E2 (n = 159 – no statistical cut-off applied). In parallel, we identified genes that were E2 responsive in the MCF-7 cells (n = 1691 p<0.05), which were not over-expressed in TAM-R vs MCF-7 cells (n = 202 – no statistical cut-off applied). The two gene sets were used to generate a Venn diagram, so genes that were both oestrogen responsive in the MCF-7 cells, and most highly expressed in 5-Aza/E2 treated TAM-Wd cells could be identified (n = 43 probes/34 genes). Using UCSC Genome Browser (http://genome.ucsc.edu), it was determined 23 of the 34 genes contained bona fide CpG islands [36] and 12 had ER-binding sites [37]. Nine of the 34 genes were found to contain both features.
(TIF)

Table S1 **CpG Island and ER-binding site status of the 34 gene candidates identified from the microarray screen.** The table shows the 34 genes derived from the microarray interrogation, and whether they contain a bona fide CpG island and/or an ER binding site within their transcription site [36], [37] (green = yes, red = no).
(TIF)
