# Supplementary figures and images for: Tamoxifen-Induced Epigenetic Silencing of Oestrogen-Regulated Genes in Anti-Hormone Resistant Breast Cancer
Source: PLoS One. 2012 Jul 10;7(7):e40466. doi: 10.1371/journal.pone.0040466 (PMC3393679; doi:10.1371/journal.pone.0040466)

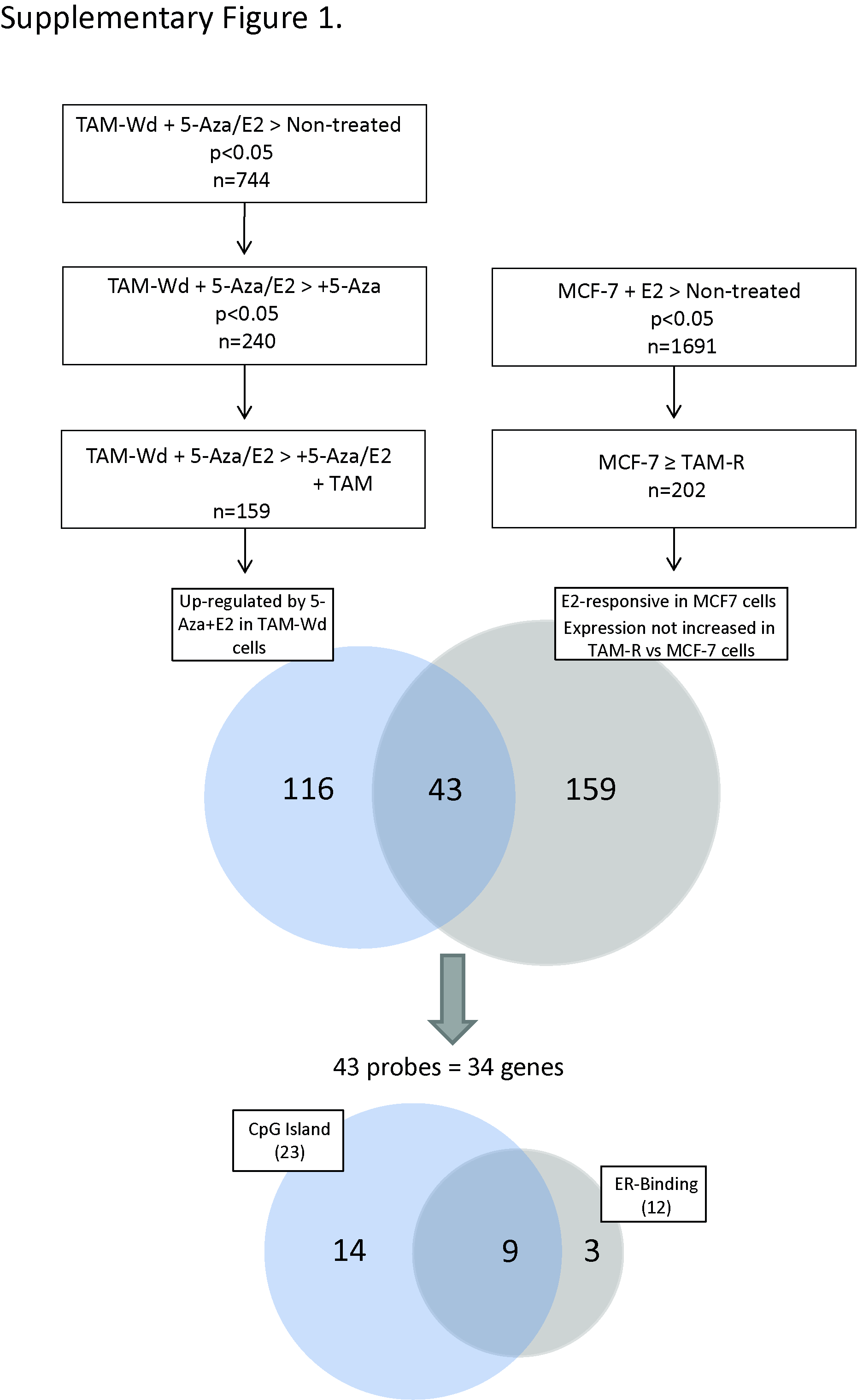

Supplement: Figure S1 — The microarray gene selection process and Venn diagram depicting ER-binding site/CpG island characteristics of the final gene candidates. The figure depicts the microarray interrogation strategy used to identify genes that could be associated with the inhibitory effect of 5-Aza/E2 co-treatment in the TAM-Wd cells (shown in Fig. 5a, 5b and 5c). Firstly, genes were selected that were most highly up-regulated in TAM-Wd cells +5-Aza/E2 vs non-treated TAM-Wd cells (n = 744 p<0.05). This gene set was further analysed to identify those genes more highly expressed in 5-Aza/E2 vs 5-Aza treated cells, to ensure the selected genes were oestrogen responsive (n = 240 p<0.05). Since we showed that TAM co-addition could block the inhibitory effect of 5-Aza/E2 treatment (Fig. 5b and 5c), we then selected genes whose expression were reduced in 5-Aza/E2+TAM treated cells vs 5-Aza/E2 (n = 159 – no statistical cut-off applied). In parallel, we identified genes that were E2 responsive in the MCF-7 cells (n = 1691 p<0.05), which were not over-expressed in TAM-R vs MCF-7 cells (n = 202 – no statistical cut-off applied). The two gene sets were used to generate a Venn diagram, so genes that were both oestrogen responsive in the MCF-7 cells, and most highly expressed in 5-Aza/E2 treated TAM-Wd cells could be identified (n = 43 probes/34 genes). Using UCSC Genome Browser (http://genome.ucsc.edu), it was determined 23 of the 34 genes contained bona fide CpG islands [36] and 12 had ER-binding sites [37]. Nine of the 34 genes were found to contain both features. (TIF) [file pone.0040466.s001.tif]

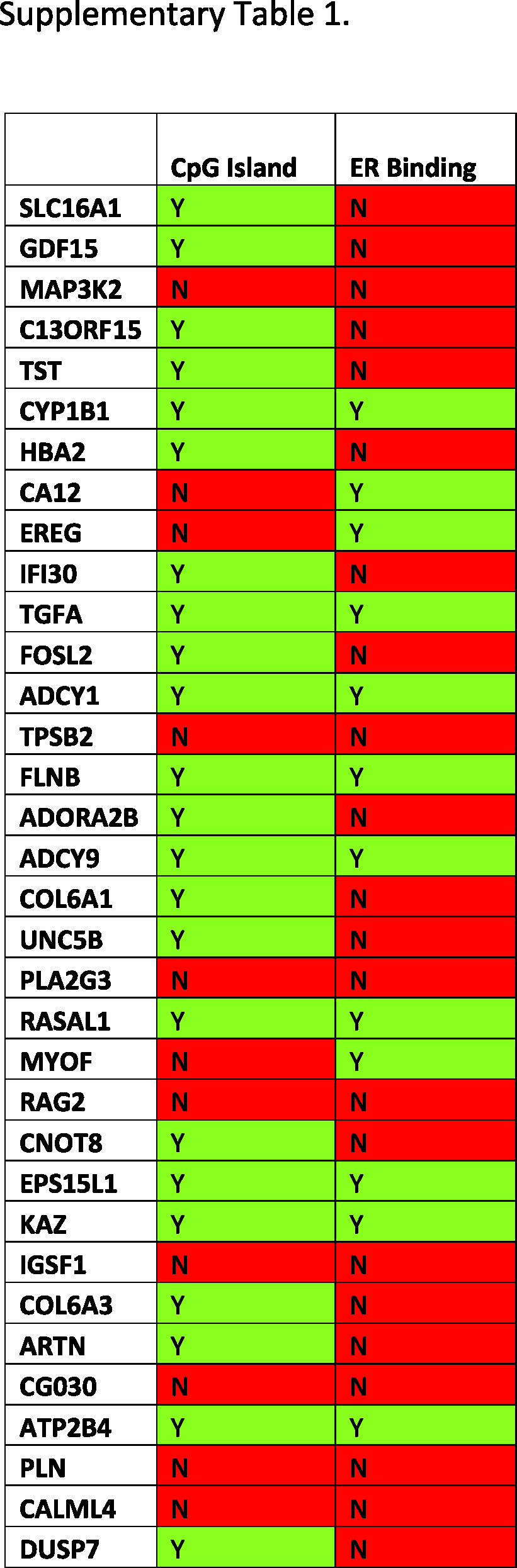

Supplement: Table S1 — CpG Island and ER-binding site status of the 34 gene candidates identified from the microarray screen. The table shows the 34 genes derived from the microarray interrogation, and whether they contain a bona fide CpG island and/or an ER binding site within their transcription site [36], [37] (green = yes, red = no). (TIF) [file pone.0040466.s002.tif]
